# Prodigiosin Sensitizes Sensitive and Resistant Urothelial Carcinoma Cells to Cisplatin Treatment

**DOI:** 10.3390/molecules26051294

**Published:** 2021-02-27

**Authors:** Lena Berning, David Schlütermann, Annabelle Friedrich, Niklas Berleth, Yadong Sun, Wenxian Wu, María José Mendiburo, Jana Deitersen, Hannah U. C. Brass, Margaretha A. Skowron, Michèle J. Hoffmann, Günter Niegisch, Jörg Pietruszka, Björn Stork

**Affiliations:** 1 Institute of Molecular Medicine I, Medical Faculty, Heinrich Heine University, 40225 Düsseldorf, Germany; lena.berning@hhu.de (L.B.); david.schluetermann@hhu.de (D.S.); annabelle.friedrich@hhu.de (A.F.); niklas.berleth@hhu.de (N.B.); yadongsunsurgery@gmail.com (Y.S.); wenxian.wu@hhu.de (W.W.); mendibur@hhu.de (M.J.M.); jana.deitersen@hhu.de (J.D.); 2 Institute of Bioorganic Chemistry, Faculty of Mathematics and Natural Sciences, Heinrich Heine University, Forschungszentrum Jülich, Stetternicher Forst, 52428 Jülich, Germany; h.brass@fz-juelich.de (H.U.C.B.); j.pietruszka@fz-juelich.de (J.P.); 3 Institute for Bio- and Geosciences 1: Bioorganic Chemistry (IBG-1), Forschungszentrum Jülich GmbH, 52428 Jülich, Germany; 4 Department of Urology, Medical Faculty, Heinrich Heine University, 40225 Düsseldorf, Germany; margaretha.skowron@med.uni-duesseldorf.de (M.A.S.); michele.hoffmann@hhu.de (M.J.H.); guenter.niegisch@med.uni-duesseldorf.de (G.N.)

**Keywords:** prodigiosin, urothelial carcinoma, cisplatin, chemoresistance, autophagy, apoptosis

## Abstract

Cisplatin-based treatment is the standard of care therapy for urothelial carcinomas. However, complex cisplatin resistance mechanisms limit the success of this approach. Both apoptosis and autophagy have been shown to contribute to this resistance. Prodigiosin, a secondary metabolite from various bacteria, exerts different biological activities including the modulation of these two cellular stress response pathways. We analyzed the effect of prodigiosin on protein levels of different autophagy- and apoptosis-related proteins in cisplatin-sensitive and -resistant urothelial carcinoma cells (UCCs). Furthermore, we investigated the effect on cell viability of prodigiosin alone or in combination with cisplatin. We made use of four different pairs of cisplatin-sensitive and -resistant UCCs. We found that prodigiosin blocked autophagy in UCCs and re-sensitized cisplatin-resistant cells to apoptotic cell death. Furthermore, we found that prodigiosin is a potent anticancer agent with nanomolar IC_50_ values in all tested UCCs. In combination studies, we observed that prodigiosin sensitized both cisplatin-sensitive and -resistant urothelial carcinoma cell lines to cisplatin treatment with synergistic effects in most tested cell lines. These effects of prodigiosin are at least partially mediated by altering lysosomal function, since we detected reduced activities of cathepsin B and L. We propose that prodigiosin is a promising candidate for the therapy of cisplatin-resistant urothelial carcinomas, either as a single agent or in combinatory therapeutic approaches.

## 1. Introduction

According to the WHO, with 570,000 new cases and 210,000 deaths in 2020, bladder cancer (BC) is one of the 10 most common cancers in the world [1]. On average, one in 100 men and one in 400 women will be diagnosed with BC during their lifetime [2]. Urothelial carcinomas (UCs) can be subdivided into non-muscle-invasive (NMIBC) and muscle-invasive bladder cancers (MIBC), the latter representing one fourth of UCs and having a risk of developing metastases [3]. Patients suffering from MIBC face a poor prognosis, with a 5-year survival rate of only 50% [4] after receiving the recommended treatment consisting of a radical cystectomy in combination with perioperative chemotherapy [2,5,6]. The outcome of patients suffering from metastatic disease, in which platin-based chemotherapy is the standard of care, have an even worse prognosis, with a long-term survival of less than 20% [7]. Even the recent introduction of immune checkpoint inhibitors in treatment algorithms did not proficiently ameliorate these results. The success of cisplatin-based therapy is limited by several factors, including the need for a sufficient renal function and the possibility of resistance development, which often necessitates second-line therapy [5]. 

Antitumor activity of platinum-containing anticancer agents has been mainly associated with their ability to form covalent linkages to nucleophilic residues of DNA bases. This leads to the formation of DNA adducts and DNA double strand breaks, resulting in the initiation of the intrinsic apoptotic pathway [8,9]. However, cisplatin can form multiple different adducts with nucleophilic residues and therefore affect multiple cellular pathways, and for this reason its exact mechanism of action remains unclear [9,10]. Therefore, cisplatin resistance mechanisms are complex and multifaceted, including an increased DNA repair capacity and anti-apoptotic ability, modifications in cellular transport and augmented anti-oxidative capacity [11,12,13]. Importantly, autophagy is another process which has been linked to the cisplatin resistance of cancer cells [14,15,16].

Autophagy is an intracellular catabolic process in which misfolded, damaged or aggregated proteins as well as whole cell organelles can be degraded and recycled. For the induction of autophagy, two kinase complexes are essential. The activation of the Unc-51-like autophagy activating kinase 1 (ULK1) protein kinase complex and the class III phosphatidylinositol 3-kinase (PtdIns3K) lipid kinase complex initiates the biogenesis of double-membraned vesicles named autophagosomes from specific subdomains of the endoplasmic reticulum (ER) [17]. After the engulfment of the cargo, the outer membrane of the autophagosome fuses with a lysosome, resulting in an autolysosome where the sequestered cargo and the inner autophagosomal membrane are degraded by lysosomal hydrolases [18]. Besides occurring at a basal level to maintain cell homeostasis in physiological conditions, autophagy can be stimulated by internal and external stimuli such as nutrient deprivation, stress conditions or chemotherapeutical anticancer treatment [19]. Since the cisplatin treatment-mediated upregulation of autophagy causes resistance and raises the threshold of efficacy, some autophagy inhibitors have been tested in clinical studies. The main focus hereby lies on chloroquine and hydroxychloroquine, which can inhibit autophagy by blocking the fusion of autophagosomes and lysosomes and are tested in combination studies with conventional chemotherapeutics [20,21]. Another autophagy-modulating compound that has previously been tested in clinical studies is obatoclax, a synthetic analogue of the natural compound prodigiosin [22,23,24].

**Figure 1:**
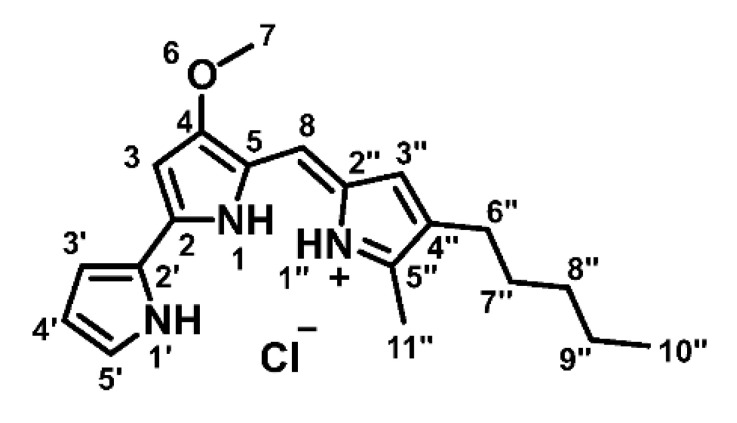
Chemical structure of prodigiosin.

Prodigiosin (Figure 1) is a deep red secondary metabolite with a tripyrrole structure, which was first extracted and characterized from the bacterium *Serratia marcescens* [25,26]. It can be found ubiquitously in various bacteria of the marine and terrestrial environment [25,27]. Prodigiosin has been shown to exert antimicrobial [28], antimalarial [29] and immunosuppressive [30] properties. In addition, prodigiosin and its synthetic analogue obatoclax have been tested in several pre-clinical and clinical trials alone or in combination with conventional chemotherapeutics as anticancer agents [22,23,24,31]. In that respect, different effects on both apoptosis and autophagy have been observed in various cancer models [31,32,33,34,35,36]. However, the molecular targets and the exact mechanisms of prodigiosin and its effects on resistant cancer cells remain unclear.

In this study, we found that prodigiosin not only decreased the viability of different cisplatin-sensitive and -resistant urothelial carcinoma cell (UCC) lines, but also sensitized them to cisplatin treatment. While autophagy was inhibited in both cisplatin-sensitive and -resistant UCCs, prodigiosin induced apoptotic cell death in cisplatin-resistant UCCs in nanomolar concentrations. Furthermore, we observed reduced activities of cathepsin B and L upon incubation with prodigiosin. Thus, we propose that treatment with prodigiosin can be a promising approach to enhance the effect of conventional chemotherapeutic drugs and potentially re-sensitize cisplatin-resistant tumors to cisplatin therapy.

## 2. Material and Methods

### 2.1. Antibodies and Reagents

Antibodies against β-actin (Sigma–Aldrich, St.Louis, MO, USA, #A5316, clone AC-74, 1:5000), LC3B (Cell Signaling Technology, Danvers, MA, USA, #2775, 1:1000), sequestosome 1 (SQSTM1) (PROGEN, Heidelberg, Germany, #GP62-C, 1:1000) and poly (ADP-ribose) polymerase (PARP)-1 (Enzo, New York, NY, USA, #BML-SA250-0050, clone C-2-10, 1:2000) were used. Isolated and purified prodigiosin was dissolved in DMSO. IRDye 680- or IRDye 800-conjugated secondary antibodies were purchased from LI-COR Biosciences (Lincoln, NE, USA, 926-68077, 926-32211 and 926-32210). Other reagents used were bafilomycin A_1_ (Sigma–Aldrich, St.Louis, MO, USA, #B1793), cisplatin (NeoCorp, Pawtucket, RI, USA, 1 mg/mL, 39021.01.00), DMSO (PanReac AppliChem, Darmstadt, Germany, #A3672 and ROTH, Karlsruhe, Germany, #7029.1), Pepstatin A (Sigma–Aldrich, St.Louis, MO, USA, #P5318), Q-VD-OPh (MP Biomedicals, Santa Ana, CA, USA, #03OPH109), staurosporine (biomol, Hamburg, Germany, #AG-CN2-0022-M005), thiazolyl blue (MTT, ROTH, Karlsruhe, Germany, #4022.3), Torin 2 (Selleckchem, Houston, TX, USA, #S2817) and Z-Phe-Phe-FMK (abcam, Cambridge, UK, #ab141386). The cathepsin activities of RT-112 and RT-112^res^ cells were measured using the fluorometric Cathepsin Activity Assay Kits (abcam, Cambridge, UK, #ab65300, #ab65302, #ab65306) according to the manufacturer’s instructions and measured with a microplate reader (BioTek, Winooski, VT, USA, Synergy Mx). 

### 2.2. Correct Identification of Natural Products

Prodigiosin (Figure 1) was produced and purified as described by Domröse et al. [37]. After column chromatography, prodigiosin was precipitated as hydrochloride as a dark red solid and a 10 mM stock in DMSO was prepared.

^1^H-NMR (600 MHz, CDCl_3_): δ [ppm] = 0.90 (t, ^3^*J*_10″,9″_ = 7.0 Hz, 3H, 10″-H), 1.32 (m_c_, 4H, 8″-, 9″-H), 1.54 (m_c_, 2H, 7″-H), 2.39 (t, ^3^*J*_6″,7″_ = 7.6 Hz, 2H, 6″-H), 2.54 (s, 3H, 11″-H), 4.00 (s, 3H, 7-H), 6.07 (d, ^4^*J*_3,1_ = 1.9 Hz, 1H, 3-H), 6.35 (m_c_, 1H, 4′-H), 6.68 (d, ^4^*J*_3″,1″_ = 2.6 Hz, 1H, 3″-H), 6.91 (ddd, ^3^*J*_3′,4′_ = 3.8 Hz, ^4^*J*_3′,5′_ = 2.4 Hz, ^5^*J*_3′,1′_ = 1.4 Hz, 1H, 3′-H), 6.95 (s, 1H, 8-H), 7.22 (m_c_, 1H, 5′-H), 12.56 (brs, 1H, 1′-NH), 12.71 (brs, 2H, 1-, 1″-NH); ^13^C-NMR (151 MHz, CDCl_3_): δ [ppm] = 12.6 (C-11″), 14.2 (C-10″), 22.6 (C-9″), 25.5 (C-6″), 29.9 (C-7″), 31.6 (C-8″), 58.9 (C-7), 93.0 (C-3), 111.9 (C-4′), 116.1 (C-8), 117.2 (C-3′), 120.8 (C-5), 122.4 (C-2′), 125.3 (C-2″), 127.1 (C-5′), 128.5 (C-3″), 128.6 (C-4″), 147.1 (C-5″), 147.8 (C-2), 165.9 (C-4).

The analytical data can be found in Supplementary Figure S1 and are in accordance to the literature [37].

### 2.3. Cell Lines and Cell Culture

All UCC lines were cultured in Dulbecco’s Modified Eagle Medium (DMEM, Thermo Fisher Scientific, Waltham, MA, USA, #41965039) containing 10% fetal bovine serum (FBS, Sigma–Aldrich, St.Louis, MO, USA, #F0804), 4.5 g/L d-glucose, 100 units/mL penicillin and 100 µg/mL Streptomycin (Thermo Fisher Scientific, Waltham, MA, USA, #15140122). All cells were cultivated and treated at 37 °C and 5% CO_2_ in a humidified atmosphere. All UCC lines have been previously described [38]. Briefly, for the generation of cisplatin-resistant cell lines, cells were treated with increasing dosages of cisplatin over several months. During cell culture, cisplatin was added to the media with every passage in concentrations of 1 µg/mL for J82, 2 µg/mL for 253J, 7 µg/mL for T24 and 12 µg/mL for RT-112 cells.

### 2.4. Cell Viability Assay

Cell viability was measured using the MTT (3-(4,5-dimethylthiazol-2-yl)-2,5-diphenyltetrazolium bromide) assay. J82, 253J, T24 and RT-112 cisplatin-sensitive or resistant cells were seeded in 96-well plates with a density of 5 × 10^4^ cells/well. One day after seeding, cells were treated with cisplatin and/or prodigiosin for 24 or 72 h. After the incubation time, MTT was added to the cells and they were incubated at 37 °C and 5% CO_2_ in a humidified atmosphere for 1 h. Upon removal of the MTT-containing medium, 100 µL DMSO per well were added for extraction of the formazan. Absorbance was measured at 570 nm and 650 nm (reference) with a microplate reader (BioTek, Winooski, VT, USA, Synergy Mx). After the subtraction of the reference value, the mean of the absorbance of the solvent control was set as 100%.

### 2.5. Immunoblotting

For SDS PAGE and western blotting, cells were harvested by scraping, pelletized at 150 rcf and 4 °C for 5 min, washed with PBS (Thermo Fisher Scientific, Waltham, MA, USA, #14190-094) and quickly frozen in liquid nitrogen. Cells were lysed in lysis buffer (20 mM Tris-HCl, 150 mM NaCl, 500 µM EDTA, 1% (*v*/*v*) Triton X-100, 1 mM Na_3_VO_4_, 10 mM NaF, 2.5 mM Na_4_P_2_O_7_, 1X protease inhibitor cocktail [Roche, Basel, Switzerland, #4693132001]) for 30 min on ice and the lysates were cleared by centrifugation at 18,000 rcf and 4 °C for 15 min. The protein concentration was determined by Bradford assay and sample buffer was added (62.5 mM Tris, 8.6% [*v*/*v*] glycerol, 2% [*w*/*v*] SDS, 33.3 µg/mL bromphenol blue, 1% [*v*/*v*] β-mercaptoethanol). Samples were heated at 95 °C for 5 min and then equal amounts of protein (25 µg) were subjected to SDS-polyacrylamide gels. After separation by SDS-PAGE, proteins were transferred to PVDF membranes (Merck, Darmstadt, Germany, #IPFL00010), blocked with 5% milk powder in TBS-T (10 mM Tris-HCl, pH 7.6, 150 mM NaCl, 0.1% Tween-20 [Sigma-Aldrich, St.Louis, MO, USA, #P1379]) and analyzed using the indicated primary antibodies followed by appropriate IRDye 680- or IRDye 800-conjugated secondary antibodies (LI-COR Biosciences, Lincoln, NE, USA). Fluorescence signals were detected using an Odyssey Infrared Imaging system (LI-COR Biosciences, Lincoln, NE, USA) and signals were quantified with Image Studio (LI-COR Biosciences, Lincoln, NE, USA).

### 2.6. Immunofluorescence

For immunofluorescence microscopy, cells were seeded in µ-Slide 8 Well (Ibidi, Graefeling, Germany, #80826). Cells were treated with 100 nM Lysotracker^TM^ Deep Red (Thermo Fisher Scientific, Waltham, MA, USA, #L12492). After the incubation time, the treatment medium was removed and replaced by DMEM without phenol red (Thermo Fisher Scientific, Waltham, MA, USA, #31053028). Representative images were acquired with an Axio Observer 7 fluorescence microscope (Carl Zeiss Microscopy, Jena, Germany) using a 40x/1.4 Oil DIC M27 Plan-Apochromat objective (Carl Zeiss Microscopy, Jena, Germany) and an ApoTome 2 (Carl Zeiss Microscopy, Jena, Germany).

### 2.7. Statistical Analysis

All IC_50_ values were calculated using GraphPad Prism 7.01. Isobologram analysis for the combined viability assays of two drugs was performed with CompuSyn [39]. The software allows for the simulation of the effects of combined drugs at any effect level and calculates the combination index (CI) of a drug combination to determine if effects are synergistic (CI < 1), additive (CI = 1) or antagonistic (CI > 1). For immunoblotting, the density of each protein band was quantified using Image Studio Lite 5.2. The density of each protein band was then divided by the average density of all bands of this protein. These ratios of the proteins of interest were then normalized to the loading control. Each normalized density ratio was divided by the mean normalized density ratio of the solvent control lane of all replicates. For all western blot analyses, the results are shown as the mean + standard deviation and the *p* values were determined for each cell line by ordinary one-way ANOVA with Dunnett´s post hoc test and are shown in the bar diagrams. For the immunofluorescence analyses, dots were quantified and analyzed using ImageJ 1.53c. The macro used for quantification is provided in the Supplementary Methods. At least 200 cells were analyzed in two biological replicates for each cell line. For comparisons between cell lines, a Student´s *t*-test was performed. For cathepsin activity assays, the results are shown as the mean + standard deviation and the *p* values were determined by ordinary two-way ANOVA with Tukey´s post hoc test and are shown in the bar diagrams. All *p* values were determined using GraphPad Prism 7.01.

## 3. Results

### 3.1. Prodigiosin Is Cytotoxic in Cisplatin-Sensitive and -Resistant RT-112 Cells

**Figure 2:**
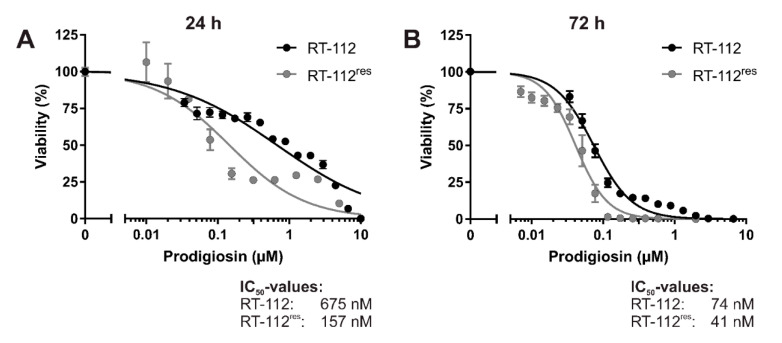
Prodigiosin is cytotoxic for cisplatin-sensitive and -resistant bladder carcinoma cells. RT-112 and RT-112^res^ cells were treated with different concentrations of prodigiosin for 24 h (**A**) or 72 h (**B**). After treatment, cell viability was measured using an thiazolyl blue (MTT) assay. Results are shown as the mean ± SEM of three independent experiments performed in triplicates for each treatment.

To analyze the effects of prodigiosin on cisplatin-resistant UCCs, we made use of RT-112 cells and the cisplatin-resistant subline RT-112^res^ [15,38]. Prodigiosin shows high cytotoxicity in RT-112 and RT-112^res^ cells with IC_50_ values of 675 nM and 157 nM after 24 h (Figure 2A) and 74 nM and 41 nM after 72 h (Figure 2B), respectively. It is noteworthy that the IC_50_ value of prodigiosin in RT-112^res^ cells was lower than in the sensitive RT-112 UCCs after both 24 h and 72 h, indicating an increased sensitivity of cisplatin-resistant cells against treatment with prodigiosin.

### 3.2. Prodigiosin Inhibits Autophagy in RT-112 Cells

**Figure 3:**
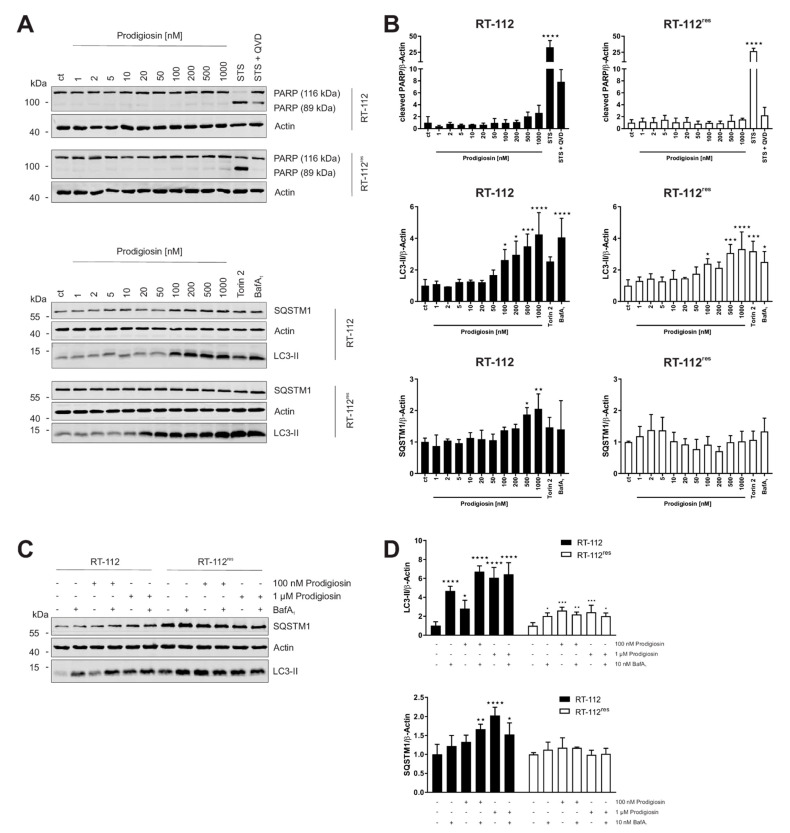
Prodigiosin modulates autophagy in RT-112 and RT-112^res^ in a concentration-dependent manner. RT-112 and RT-112^res^ cells were treated with the indicated concentrations of prodigiosin or 2.5 µM STS ± 10 µM QVD, 10 nM bafilomycin A_1_ (BafA_1_) or 250 nM Torin 2. After 6 h, the cells were lysed and cellular lysates were immunoblotted for the indicated proteins. (**A**,**C**) One representative immunoblot is shown for each experiment. (**B**,**D**) The densities of the bands of each protein of at least three independent experiments were quantified and normalized to actin. The mean of the solvent control of RT-112 (black bars) and RT-112^res^ (white bars) was set as 1 for each protein. Bars represent the means + SD. *p* values were determined by ordinary one-way ANOVA with Dunnett´s post hoc test. * *p* < 0.05; ** *p* < 0.01; *** *p* < 0.001; **** *p* < 0.0001. PARP: poly (ADP-ribose) polymerase; SQSTM1: sequestosome 1; LC3: light chain 3.

Since increased anti-apoptotic capacity and upregulated autophagy are both associated to cisplatin resistance in UCCs, our next aim was to investigate the effect of prodigiosin in RT-112 and RT-112^res^. Apoptosis induction was determined by immunoblot analysis of the cleavage of the caspase-3 substrate poly (ADP-ribose) polymerase (PARP). After a 6 h incubation with prodigiosin, no significant change in PARP cleavage was observed in RT-112 and RT-112^res^ cells (Figure 3A). In contrast, prodigiosin showed significant effects on the levels of proteins associated with autophagy starting at a concentration of 100 nM (Figure 3A,B). Increased levels of the ubiquitin-like protein microtubule-associated proteins 1A/1B light chain 3 (LC3) can be associated with induced autophagy, but also occur when the autophagic machinery is inhibited in later steps. In this case, an autophagosome-bound form of LC3 (LC3-II) accumulates due to the absence of lysosomal degradation. This is illustrated by the effect of the vacuolar-type H^+^-ATPase (V-ATPase) inhibitor bafilomycin A_1_ (BafA_1_), which inhibits the acidification of the lysosome and thus prevents the degradation of engulfed cargo and LC3-II by lysosomal proteases such as cathepsins [40]. To further characterize the autophagy-modulating properties of prodigiosin, sequestosome 1 (SQSTM1) levels were investigated. SQSTM1, also known as ubiquitin-binding protein p62, binds cargo proteins to selectively target them for autophagic degradation. The concentration-dependent accumulation of SQSTM1 in RT-112 in combination with the elevated LC3-II levels suggests an inhibition of the autophagic process. In contrast, there is no significant change of—the apparently elevated—SQSTM1 levels in RT-112^res^ after treatment with up to 1 µM prodigiosin while LC3-II levels are increased (Figure 3A,B). 

### 3.3. Autophagy-Related Protein Are Upregulated in RT-112^res^ Cells

Next, we aimed at investigating whether cisplatin resistance affects the basal expression levels of LC3-II and SQSTM1 and the functional relationship of prodigiosin and BafA_1_. As determined by immunoblotting, the basal levels of both LC3-II and SQSTM1 are upregulated in RT-112^res^ cells compared to RT-112 cells (Figure 3C), which matches with previous observations [15] and underlines the role of autophagy in the resistance mechanism of UCCs against cisplatin. LC3-II levels significantly increase in sensitive and resistant cells after treatment with prodigiosin and BafA_1_, but effects are not additive. The latter observation indicates that autophagic flux is indeed blocked. Of note, LC3-II levels in RT-112^res^ cells increase only twofold upon prodigiosin or BafA_1_ treatment compared to untreated cells, which might be explained by higher basal levels and lower capacity of inducible autophagy in this cell line. SQSTM1 levels increased with prodigiosin and BafA_1_ treatment alone or in combination in RT-112, but effects are not additive, whereas SQSTM1 levels are not affected in RT-112^res^ (Figure 3C,D). Taken together, these results suggest that RT-112^res^ cells likely have a higher capacity for basal autophagy but that autophagy can still be modulated by prodigiosin treatment.

### 3.4. Prodigiosin Induces Apoptotic Cell Death in RT-112^res^

**Figure 4:**
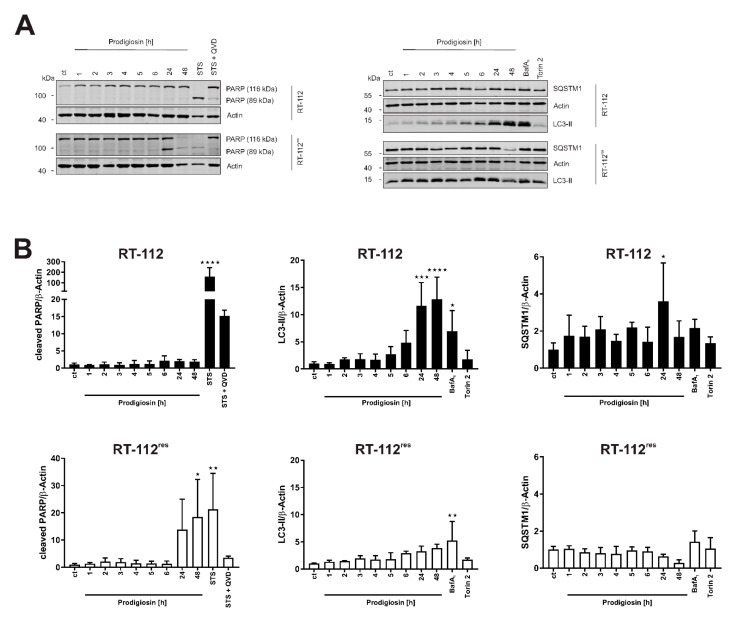
Prodigiosin blocks autophagy in RT-112 and RT-112^res^ and induces apoptosis in RT-112^res^ in a time-dependent manner. (**A**) RT-112 and RT-112^res^ cells were treated with 100 nM prodigiosin for the indicated periods of time or with 2.5 µM STS ± 10 µM QVD, 10 nM BafA1 or 250 nM Torin 2 for 24 h. After the incubation, cells were lysed, and cellular lysates were immunoblotted for the indicated proteins. One representative immunoblot is shown for each experiment. (**B**) The densities of bands of each protein of at least three independent experiments were quantified and normalized to actin. The mean of the solvent control of RT-112 (black bars) and RT-112^res^ (white bars) was set as 1 for each protein. Bars represent the means + SD. *p* values were determined by ordinary one-way ANOVA with Dunnett´s post hoc test. * *p* < 0.05; ** *p* < 0.01; *** *p* < 0.001; **** *p* < 0.0001. PARP: poly (ADP-ribose) polymerase; SQSTM1: sequestosome 1; LC3: light chain 3.

In a next step, the time-dependency of the effect of prodigiosin on apoptotic and autophagic protein markers was investigated. For these experiments, we chose a concentration of 100 nM of prodigiosin as the lowest concentration that significantly increased LC3-II levels in both RT-112 and RT-112^res^. In RT-112, there is no significant increase in cleaved PARP levels even after 48 h whereas in RT-112^res^, there is a time-dependent and significant increase in PARP cleavage after incubation with prodigiosin (Figure 4A,B), indicating apoptotic cell death in cisplatin-resistant UCCs upon this treatment. The decreased PARP and actin levels after 48 h indicate that cells are already in the late phases of apoptosis at this time point. The time-dependent accumulation of LC3-II can be seen in both cell types and is again more prominent in sensitive cells. The effects on SQSTM1 are distinct in cisplatin-sensitive and -resistant UCCs. Whereas SQSTM1 accumulates in RT-112 over time, in RT-112^res^ there is a decrease in protein level after 24 h and 48 h treatment with prodigiosin (Figure 4A,B). Taken together, these results suggest that prodigiosin blocks autophagy in cisplatin-sensitive RT-112 cells, but does not induce apoptosis. In RT-112^res^ cells, apoptotic cell death is induced, whereas SQSTM1 levels decrease despite the blocked autophagy.

### 3.5. Prodigiosin Synergistically Increases Cisplatin-Mediated Cytotoxicity in RT-112 and RT-112^res^ UCCs

**Figure 5:**
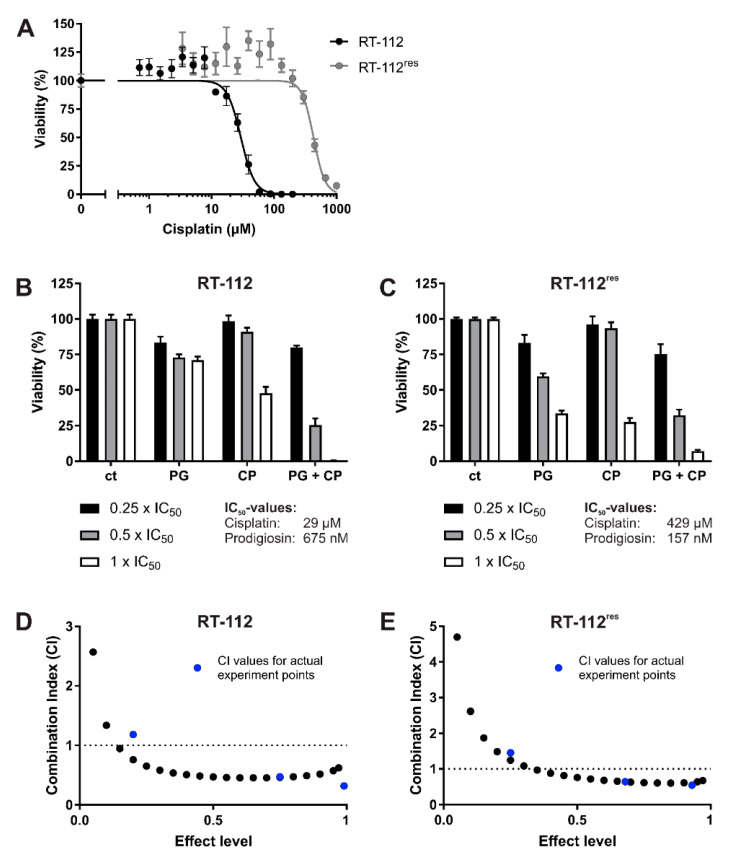
Prodigiosin increases cisplatin-mediated cytotoxicity in RT-112 and RT-112^res^ cells after 24 h. RT-112 and RT-112^res^ cells were treated with different concentrations of (**A**) cisplatin (CP) alone or (**B**,**C**) prodigiosin (PG) or CP alone or in combination for 24 h. For combinatory analysis, 0.25x, 0.5x or 1x of the IC_50_ values of the single substances in RT-112 (**B**) and RT-112^res^ (**C**) were used. After treatment, cell viability was measured using an MTT assay. Results are shown as the mean ± SEM of three independent experiments performed in triplicates for each treatment. The combination index (CI) for different fractions affected of RT-112 (**D**) and RT-112^res^ (**E**) was calculated using the software CompuSyn (black dots). CompuSyn uses algorithms to extrapolate CI values for any effect level from the CI values of actual experiment points (blue dots). Synergism (CI < 1), additivism (CI = 1) and antagonism (CI > 1) can thereby be determined.

Since modifications in both autophagy and apoptosis seem to contribute to cisplatin resistance in UCCs, we hypothesized that targeting these processes with prodigiosin might be beneficial to increase the efficiency of cisplatin treatment in BC. To analyze whether prodigiosin can have a synergistic effect on cytotoxicity together with cisplatin, we examined the effect of prodigiosin and cisplatin alone (Figure 5A) and in combination on these cell lines and performed isobologram analyses. For this, the Chou–Talalay method is the most impactful approach to quantify synergy [41]. By applying this method, it is possible to determine the combination index (CI) values for drug combinations out of a small number of data points while still receiving the maximal amount of useful information via computer simulation. Chou and Talalay therefore propose to use multiples and dividers of the respective IC_50_ concentrations of each drug and carry out the experiment at an equipotency ratio so that the contributions of the effects of each drug to the combination is roughly equal [42]. Here, cells were treated with concentrations in the range of 0.25, 0.5 or 1 times the IC_50_ values of the respective cell lines and substances. For CI plots, the CI values (calculated from actual experiment points and extrapolated) are plotted against the effect level (an effect level of 0.99 represents a reduction of cell viability by 99%). CI values < 1 are considered synergistic, whereas a CI value around 1 represents additive effects and CI values > 1 indicate antagonistic effects of the applied drugs [38]. In both RT-112 and RT-112^res^, the treatment with IC_50_ concentrations of prodigiosin and cisplatin combined leads to a nearly complete decrease in cell viability (Figure 5B,C). The combination index plot shows synergistic effects in both cell types after 24 h of treatment with a combination of prodigiosin and cisplatin especially in higher administered concentrations (Figure 5D,E). After 72 h of incubation, prodigiosin synergistically sensitizes RT-112 cells to cisplatin treatment at most effect levels. In RT-112^res^ cells, we observed synergistic effects at low- to mid-range effect levels, whereas the combination of prodigiosin and cisplatin possesses a rather additive effect at higher effect levels (Figure S2).

### 3.6. Treatment with Prodigiosin Overcomes Apoptosis Resistance in RT-112^res^

**Figure 6:**
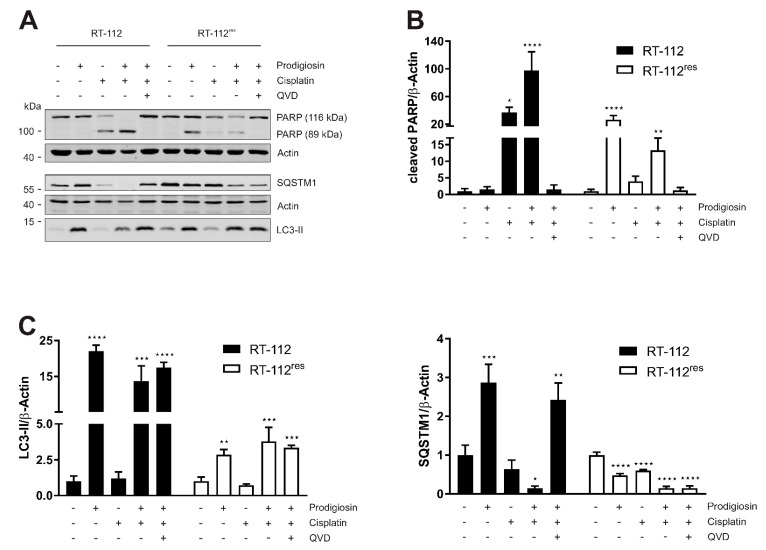
The combination of prodigiosin and cisplatin modulates apoptosis and autophagy in RT-112 and RT-112^res^. RT-112 and RT-112^res^ cells were treated with IC_50_ concentrations of prodigiosin and cisplatin alone or in combination in absence or presence of 10 µM QVD. After 24 h, the cells were lysed and cellular lysates were immunoblotted for the indicated proteins. (**A**) One representative immunoblot is shown for each experiment. The densities of bands of cleaved PARP (**B**) or LC3-II and SQSTM1 (**C**) of at least three independent experiments were quantified and normalized to actin. The mean of the solvent control of each cell line was set as 1 for each protein. Bars represent the means + SD. *p* values were determined by ordinary one-way ANOVA with Dunnett´s post hoc test. * *p* < 0.05; ** *p* < 0.01; *** *p* < 0.001; **** *p* < 0.0001. PARP: poly (ADP-ribose) polymerase; SQSTM1: sequestosome 1; LC3: light chain 3.

Subsequently, we wanted to confirm the results of the cell viability assay by the western blot analysis of autophagic and apoptotic marker proteins. IC_50_ concentrations obtained from viability assays performed after 24 h of treatment were used for each substance and cell line, respectively. Cisplatin induced a significant increase in PARP cleavage in sensitive UCCs but not in RT-112^res^, confirming the described mechanism of cytotoxicity of cisplatin [8] and the resistance to these mechanisms of RT-112^res^ cells. In contrast to RT-112 cells, in which prodigiosin has no effect on PARP cleavage, prodigiosin treatment of RT-112^res^ cells significantly induces apoptosis, which can be rescued by the caspase inhibitor QVD (Figure 6A,B). Treatment with prodigiosin, but not with cisplatin, led to a significant increase in LC3-II levels in both RT-112 and RT-112^res^. Again, the effect of prodigiosin treatment on SQSTM1 levels was contrary in resistant and sensitive UCCs. Whereas in RT-112, prodigiosin treatment alone or in combination with cisplatin and QVD led to an increase in SQSTM1, indicating that autophagy was blocked in these cells, in RT-112^res^ cells, the SQSTM1 levels decreased significantly upon all treatment regimens (Figure 6A,C). These effects can be obtained in the cisplatin-resistant cell line RT-112^res^ in lower concentrations than in the sensitive parental cells. Mostly, these effects are synergistic at higher effect levels, which is beneficial for cancer treatment. Collectively, it seems that the application of prodigiosin can sensitize RT-112 UCCs to cisplatin treatment.

### 3.7. Prodigiosin Treatment Alters Cathepsin Activity

**Figure 7:**
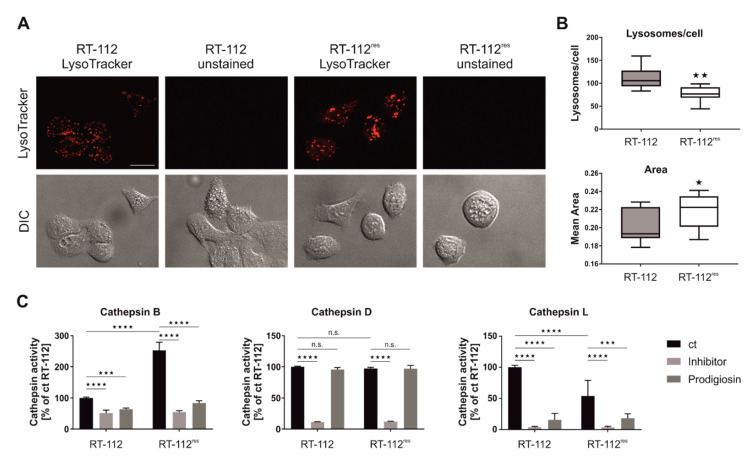
Prodigiosin treatment reduces cathepsin B and L activity in RT-112 and RT-112^res^. (**A**,**B**) RT-112 and RT-112^res^ cells in chambered coverslips were treated with 100 nM LysoTracker^TM^ Deep Red for 30 min. (**A**) Representative sections are depicted and (**B**) the number and area of lysosomes of 10 representative images from two biological replicates for each cell line were quantified using ImageJ 1.53c. *p* values were determined by Student´s *t*-test. Scale bar: 20 µm. (**C**) RT-112 and RT-112^res^ cells were treated with 100 nM prodigiosin. After 24 h, the cells were lysed and cathepsin assays were performed according to the manufacturer’s instructions. For cathepsin B and L assays, 20 µM Z-Phe-Phe-FMK and for cathepsin D assays, 0.1 µM pepstatin A were used as inhibitor control. The fluorescence of duplicates for each treatment of three independent experiments was measured and the mean of the DMSO control of RT-112 was set as 100%. Bars represent the means + SD. *p* values were determined by two-way ANOVA with Tukey´s multiple comparisons post hoc test. * *p* < 0.05; ** *p* < 0.01; *** *p* < 0.001; **** *p* < 0.0001.

It has previously been suggested that lysosomal functions are altered in cisplatin-resistant cell lines [43]. We next analyzed the lysosomal compartment of RT-112 and RT-112^res^ cells by staining with LysoTracker. We observed reduced numbers but increased sizes of lysosomes in RT-112^res^ cells (Figure 7A,B), indicating that acquired cisplatin resistance is accompanied with an altered lysosomal compartment in this cellular model. Next, we wanted to know whether prodigiosin treatment affects the activity of cathepsins, which play a key role among lysosomal proteases [44]. We employed assays for the cathepsins B, D and L, and found that especially the activities of the cysteine cathepsins B and L are significantly reduced in both RT-112 and RT-112^res^ cells upon prodigiosin treatment (Figure 7C). In contrast, the activity of the aspartic protease cathepsin D was apparently not affected by prodigiosin, but this might be explained by the observation that this protease shows residual activity under a neutral pH [45]. Taken together, we hypothesize that the observed effect of prodigiosin on RT-112^res^ cells might be caused by an altered lysosomal compartment in combination with the effects on cathepsins.

### 3.8. Treatment with Prodigiosin Synergistically Sensitizes Various UCCs to Cisplatin-Mediated Cell Death

**Figure 8:**
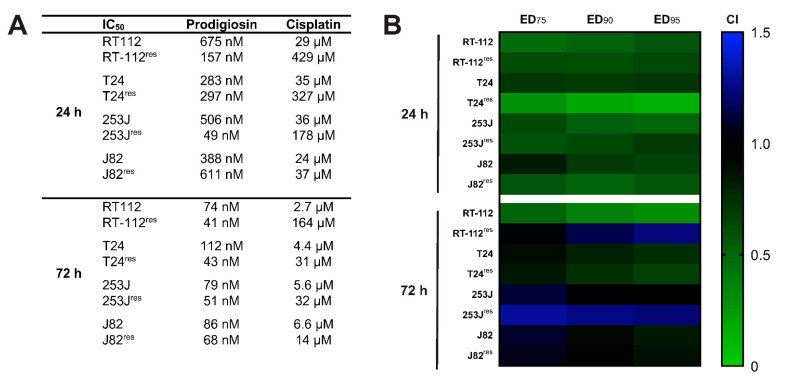
Prodigiosin increases cisplatin-mediated cytotoxicity in sensitive and -resistant bladder carcinoma cells. RT-112, RT-112^res^, T24, T24^res^, 253J, 253J^res^, J82 and J82^res^ cells were treated with different concentrations of prodigiosin and cisplatin alone or in combination for 24 or 72 h. After treatment, cell viability was measured using an MTT assay. (**A**) IC_50_ values were calculated using GraphPadPrism using the results of three independent experiments performed in triplicates for each treatment. (**B**) For combinatory analysis, cells were treated with multiples of the IC_50_ concentrations of prodigiosin and cisplatin alone or in combination. CI values were calculated for different effective doses (ED) using the software CompuSyn. CompSyn uses algorithms to extrapolate CI values for any effect level from the CI values of actual experiment points. Synergism (CI < 1; green), additivism (CI = 1; black) and antagonism (CI > 1; blue) can thereby be determined.

In a next step, we wanted to confirm our results in additional UCC lines to address the heterogeneity of the disease. Similar to RT-112 UCCs, we first determined the IC_50_ values of prodigiosin and cisplatin for each cisplatin-sensitive and -resistant cell line (Figure 8A). The decreasing differences in the IC_50_ values of cisplatin in cisplatin-sensitive and -resistant cells reflect the different permanent cisplatin concentrations in the culture media of RT-112^res^, T24^res^, 253J^res^ and J82^res^, which are 12 µg/mL (39.9 µM), 7 µg/mL (23.3 µM), 2 µg/mL (6.6 µM) and 1 µg/mL (3.3 µM), respectively. Of note, IC_50_ values of prodigiosin are lower in the resistant cell lines compared to the cisplatin-sensitive parental cells except for T24^res^ and J82^res^ cells after 24 h. Based on these data, we repeated the above-described combination experiments and isobologram analyses. After 24 h treatment, CI values at different effective doses (EDs) were synergistic in all cisplatin-sensitive and -resistant cell lines, whereas after 72 h of treatment the combination effects vary between cell lines (Figure 8B). Whilst in T24 and T24^res^, effects were synergistic at all displayed EDs, the combination was rather additive to slightly synergistic in 253J, J82 and J82^res^. In RT-112^res^ cells, CI values in the lower antagonistic range were obtained for higher effective doses, and antagonism was observed in 253J^res^ cells for the 72 h incubation. Of note, in all tested cell lines these effects can be seen after treatment with prodigiosin in nanomolar ranges. Due to low nanomolar IC_50_ values in cisplatin-resistant UCCs, a monotherapy with prodigiosin in a second-line treatment of cancers with acquired cisplatin resistance could also be an interesting therapeutical approach, which naturally requires further investigations. Taken together, these findings present prodigiosin as a promising candidate for the therapy of cisplatin-resistant bladder cancer. 

## 4. Discussion

Bladder cancer is one of the ten most common cancers and patients face a poor prognosis due to limited treatment options. Cisplatin-based combination treatment is recommended as first-line therapy, but success is often limited by harmful side effects and chemoresistance [2,5,6]. Natural compounds can be a valuable source for lead structures that increase cisplatin efficacy or overcome resistance mechanisms. In this study, we characterized the effects of the bacterial tripyrrole prodigiosin on UCC lines. We observed that prodigiosin blocks autophagy in both cisplatin-sensitive and -resistant UCCs, but preferentially induces apoptotic cell death in cisplatin-resistant UCCs. We also found that basal levels of autophagy-related proteins increased with cisplatin resistance. This stands in line with the observations by our own group [15] and by Ojha et al. [46] who demonstrated that cell protective autophagy was upregulated in a cisplatin-treated T24 UCC line and that the inhibition of autophagy through chloroquine increased the cytotoxic effect of cisplatin. 

Effects of prodigiosin on autophagy are discussed controversially in the literature. On the one hand, the inhibition of the autophagic process has been described via different mechanisms such as the extracellular signal-regulated kinase pathway or by blocking autophagosome­–lysosome fusion and lysosomal cathepsin maturation [33,34,36]. On the other hand, other studies report that prodigiosin treatment led to the induction of autophagic cell death [32,47]. The discrepancies between these observations about prodigiosin suggest that effects might depend strongly on the type of cancer. In our hands, prodigiosin rather reveals an autophagy-inhibitory function in both sensitive and -resistant UCCs as determined by autophagic flux assays using bafilomycin A_1_. Under physiological conditions, SQSTM1 levels are relatively low due to continuous degradation by autophagy. Matching with our observations, elevated SQSTM1 levels have strongly been indicated to be involved in resistance to platinum-based cancer therapy [48]. Notably, we observe a decrease in SQSTM1 levels upon prolonged treatment with prodigiosin (i.e., 24–48 h). However, this does not necessarily indicate that SQSTM1 becomes degraded by a (macro-)autophagy-dependent pathway and might be mediated by alternative mechanisms in cells destined for death. For example, it has been described that SQSTM1 can be cleaved by caspases 6 and 8, respectively [49]. 

Like in cisplatin-resistant UCCs, prodigiosin has also been shown to induce apoptotic cell death in various other cell models [33,50,51,52,53]. Since apoptosis in resistant UCCs could be induced by prodigiosin, but not by cisplatin, it seems that prodigiosin is able to partially overcome cisplatin resistance by re-establishing apoptotic pathways. Generally, there is a strong relationship between autophagy and cisplatin resistance. Recently, Gąsiorkiewicz et al. reviewed autophagy-modulating compounds that chemosensitize for cisplatin in cancer therapy [54]. Among these compounds are classical autophagy inhibitors, compounds with specific autophagy-related targets, and natural compounds. It appears that both the inhibition of the cyto-protective functions and the induction of death-promoting functions of autophagy can be therapeutically beneficial. We observe an altered lysosomal compartment in RT-112^res^ cells, and prodigiosin treatment clearly affects cathepsin activity. Of note, we found that cathepsin D is less affected by prodigiosin, and this cathepsin has been reported to be partially active at neutral pH [45]. We speculate that prodigiosin influences intralysosomal cathepsin activation, and apparently the lysosomal structure in cisplatin-resistant RT-112^res^ cells favors this effect. It has been described that prodigiosin can mediate a rise of the lysosomal pH with a concomitant non-organelle-specific increase in acidity of the cytosol [55]. However, Zhao et al. reported that prodigiosin inhibits lysosomal activity by downregulating mRNA and protein levels of cathepsins without affecting the pH [36]. Accordingly, further studies have to assess the actual target of prodigiosin mediating this effect.

The National Cancer Institute has tested prodigiosin against a suite of around 60 cancer cell lines with an average IC_50_ of 2.1 µM [31]. In our experiments, prodigiosin had IC_50_ values in the nanomolar range in all tested UCCs. It is noteworthy that there was a tendency to lower IC_50_ values in cisplatin-resistant cells compared to their sensitive precursors. Furthermore, we found mostly synergistic or additive cytotoxic effects when prodigiosin was combined with cisplatin, especially at higher effect levels. As specified by Chou [42], it is important to note that synergism and antagonism can be different at different dose or effect levels. While for chronic diseases synergism at low dose or effect levels is important, for the treatment of infectious diseases or cancer therapies, synergism at high effect levels is much more therapeutically relevant.

In addition, selective synergism against the target and antagonism toward the host is also of practical importance to enlarge the therapeutic window. Several studies suggest desirable clinical properties of prodigiosin. Using the Ames and micronucleus test, Guryanov et al. could show that prodigiosin exhibited no significant genotoxic potential [56]. Furthermore, prodigiosin shows no adverse effects in a combination study with 5-fluorouracil in colorectal cancer in mice while sensitizing cancer cells to 5-fluorouracil [36], and it has been shown to exert higher toxicity in A549 lung carcinoma cells than in primary small airway epithelial cells [57]. For therapeutic application, it has to be considered whether the concentrations for synergism are achievable in the body [42]. Prodigiosin meets Lipinski´s rule of five which predicts the oral bioavailability of drugs [58,59], but certainly, further evaluations of the bioavailability of prodigiosin in the target tissue are required.

To our knowledge, this is the first study that investigates the influence of prodigiosin in combination with cisplatin in chemoresistant bladder cancer cells. Overall, we propose that treatment with prodigiosin can enhance the efficacy of cisplatin-based chemotherapy in UCCs to resensitize resistant tumors and is worthwhile further investigations. 

## Data Availability

Not applicable.

## Supplementary Materials

The following are available online, Figure S1: ^1^H- and ^13^C-NMR-spectra of Prodigiosin (CDCl_3_, 600 MHz and 151 MHz, respectively), Figure S2: Prodigiosin increases cisplatin-mediated cytotoxicity in RT-112 and RT-112^res^ cells after 72 h, Supplementary Methods: Macro for quantification of LysoTracker-stained lysosomes.

## Sample Availability

Samples of the compounds are not available from the authors.

## Abbreviations

BafA_1_, bafilomycin A_1_; Bcl-2, B-cell lymphoma 2; BC, bladder cancer; CI, combination index; ED, effective dose; ER, endoplasmic reticulum; ERK, extracellular signal-regulated kinase; (MAP1)LC3, (microtubule-associated proteins 1A/1B) light chain 3; IC_50_, half maximal inhibitory concentration; MIBC, muscle-invasive bladder cancer; MTT, 3-(4,5-Dimethylthiazolyl-2)-2,5-diphenyl-2H-tetrazoliumbromid; NMIBC, non-muscle-invasive bladder cancer; PARP, poly (ADP-ribose) polymerase; PtdIns3K, phosphatidylinositol 3-kinase; SQSTM1, sequestosome 1; STS, staurosporine; UC, urothelial carcinoma; UCC, urothelial carcinoma cell; ULK1, Unc-51 like autophagy activating kinase 1; V-ATPase, vacuolar-type H+-ATPase
