# Supplementary material for: Prodigiosin Sensitizes Sensitive and Resistant Urothelial Carcinoma Cells to Cisplatin Treatment
Source: Molecules. 2021 Feb 27;26(5):1294. doi: 10.3390/molecules26051294 (PMC7957586; doi:10.3390/molecules26051294)
Supplement: Supplementary file 1 [file molecules-26-01294-s001.pdf]

Supplementary Material

Prodigiosin Sensitizes Sensitive and Resistant Urothelial Carcinoma Cells to Cisplatin Treatment

Lena Berning [1], David Schlütermann [1], Annabelle Friedrich [1], Niklas Berleth [1], Yadong Sun [1], Wenxian Wu [1], María José Mendiburo [1], Jana Deitersen [1], Hannah U. C. Brass [2,3], Margaretha A. Skowron [4], Michèle J. Hoffmann [4], Günter Niegisch [4], Jörg Pietruszka [2,3] and Björn Stork [1,*]

[1] Institute of Molecular Medicine I, Medical Faculty, Heinrich Heine University, 40225 Düsseldorf, Germany; lena.berning@hhu.de (L.B.); david.schluetermann@hhu.de (D.S.); annabelle.friedrich@hhu.de (A.F.); niklas.berleth@hhu.de (N.B.); yadongsunsurgery@gmail.com (Y.S.); wenxian.wu@hhu.de (W.W.); mendibur@hhu.de (M.J.M.); jana.deitersen@hhu.de (J.D.)

[2] Institute of Bioorganic Chemistry, Faculty of Mathematics and Natural Sciences, Heinrich Heine University, Forschungszentrum Jülich, Stetternicher Forst, 52428 Jülich, Germany; h.brass@fz-juelich.de (H.U.C.B.); j.pietruszka@fz-juelich.de (J.P.)

[3] Institute for Bio- and Geosciences 1: Bioorganic Chemistry (IBG-1), Forschungszentrum Jülich GmbH, 52428 Jülich, Germany

[4] Department of Urology, Medical Faculty, Heinrich Heine University, 40225 Düsseldorf, Germany; margaretha.skowron@med.uni-duesseldorf.de (M.A.S.); michele.hoffmann@hhu.de (M.J.H.); guenter.niegisch@med.uni-duesseldorf.de (G.N.)

* Corresponding author: bjoern.stork@hhu.de; Tel.: +49 (0)211 81 11954, Fax: +49 (0)211 81 11611954

Supplementary Figures S1 and S2

Supplementary Methods

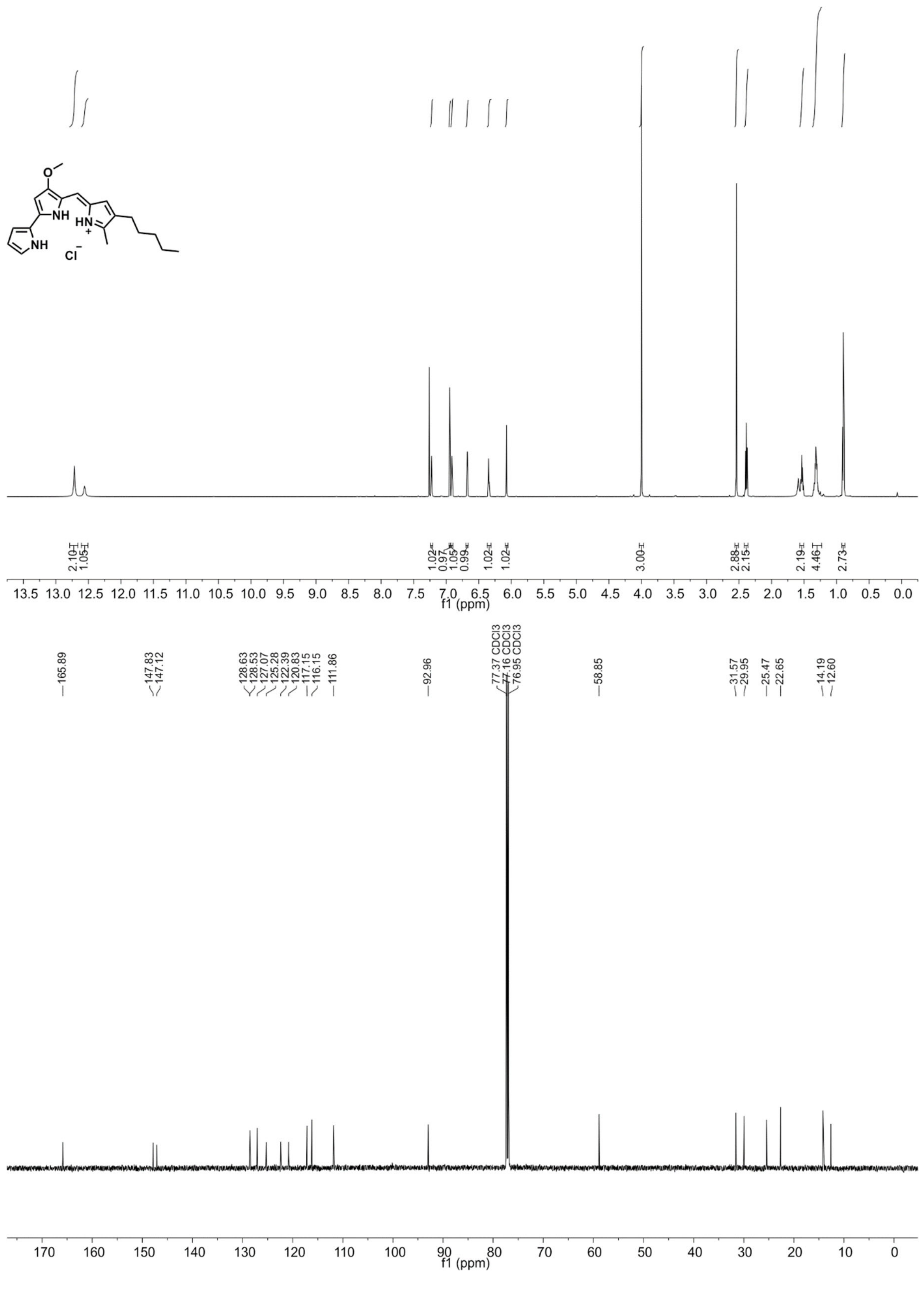

Figure S1:

^{1}H- and ^{13}C-NMR-spectra of Prodigiosin ($CDCl_3$, 600 MHz and 151 MHz, respectively).

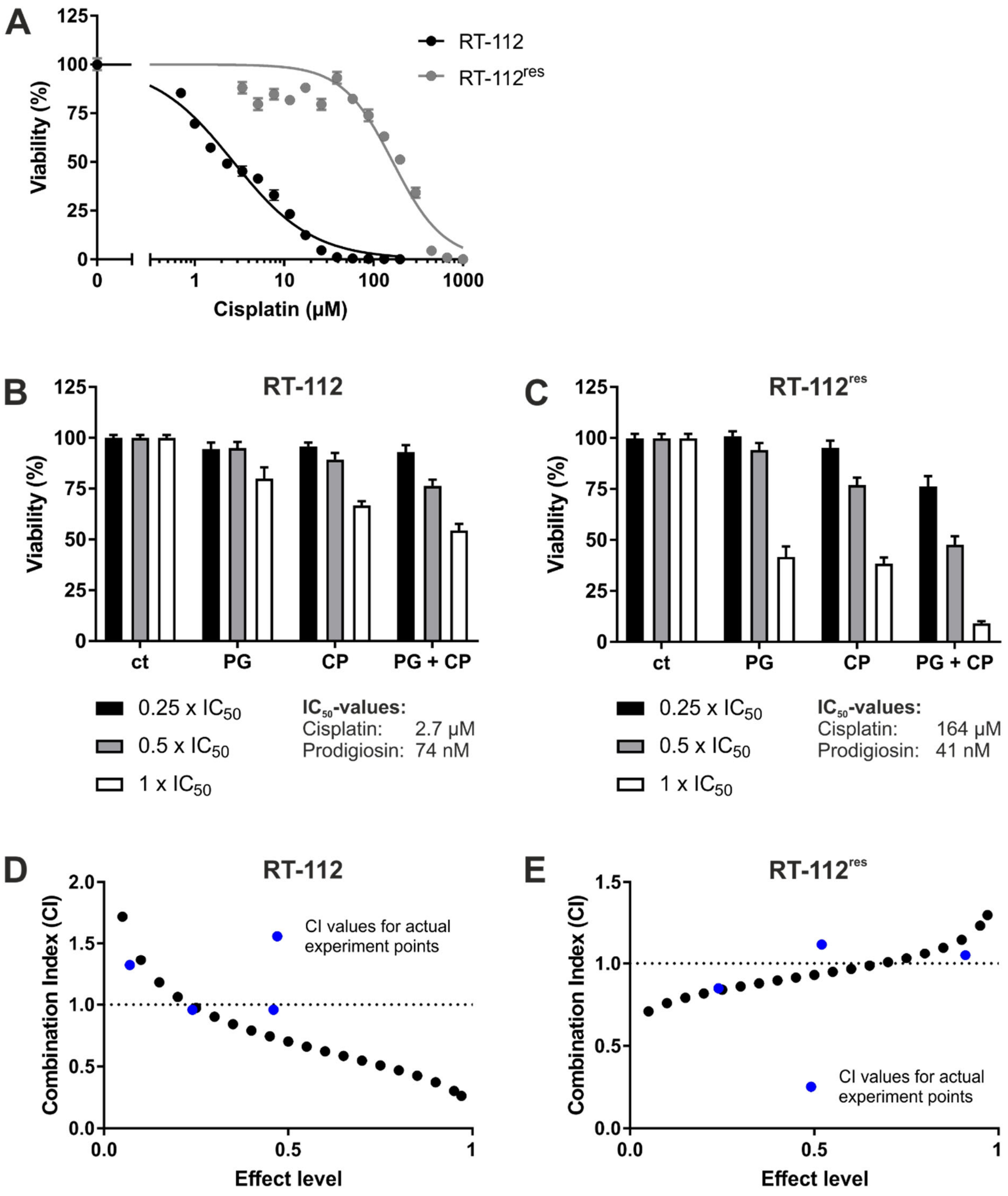

Figure S2:

Prodigiosin increases cisplatin-mediated cytotoxicity in RT-112 and RT-112res cells after 72 h. RT-112 and RT-112res cells were treated with different concentrations of (**A**) cisplatin (CP) alone or (**B+C**) prodigiosin (PG) or CP alone or in combination for 72 h. For combinatory analysis 0.25x, 0.5x or 1x of the IC_{50} values of the single substances in RT-112 (B) and RT-112res

(C) were used. After treatment, cell viability was measured using an MTT assay. Results are shown as mean ± SEM of three independent experiments performed in triplicates for each treatment. The Combination Index (CI) for different fractions affected of RT-112 (**D**) and RT-112res (**E**) was calculated using the software CompuSyn (black dots). CompuSyn uses algorithms to extrapolate CI values for any effect level from the CI values of actual experiment points (blue dots). Synergism (CI < 1), additivism (CI = 1) and antagonism (CI > 1) can thereby be determined.

Supplementary Methods

For quantification of LysoTracker-stained lysosomes, the following Macro was used (ImageJ 1.53c):

```
dir1 = getDirectory("INPUT ");
dir2 = getDirectory("OUTPUT ");
setBatchMode(true);
list = getFileList(dir1);
for (i=0; i<list.length; i++)
{
open(dir1+list[i]);
title = File.nameWithoutExtension ;
//save channels
run("Options...", "iterations=1 count=1 black do=Nothing");
run("Z Project...", "projection=[Max Intensity]");
run("Duplicate...", " ");
saveAs("Tiff", dir2+title+"_lysosomes_roi.tif");
close();
run("8-bit");
saveAs("Tiff", dir2+title+"_lysosomes.tif");
//process and count lysosomes
run("Convoluted Background Subtraction", "convolution=Median radius=10");
run("Enhance Contrast...", "saturated=0.01 normalize");
run("Auto Threshold", "method=IsoData white");
run("Adjustable Watershed", "tolerance=0.5");
saveAs("Tiff", dir2+title+"_lysosomes_processed.tif");
run("Analyze Particles...", "size=0.01-Infinity show=Outlines exclude clear summarize add");
saveAs("Tiff", dir2+title+"_lysosomes_drawing.tif");
close();
//select all ROIs in ROI manager
count=roiManager("count");
array=newArray(count);
for(f=0; f<count;f++)
array[f] = f;
roiManager("Select", array);
close();
close();
//transfer ROIs to original image and measure intensities
open(dir2+title+"_lysosomes_roi.tif");
roiManager("Show All");
roiManager("Measure");
run("8-bit");
saveAs("Tiff", dir2+title+"_lysosomes_roi.tif");
//export to excel file on desktop
```

```
selectWindow("Results");
run("Read and Write Excel");
close();
}
setBatchMode(false);
//save summary sheet
selectWindow("Summary");
saveAs("Text", dir2+"Summary.txt");
run("Close All")
showMessage("You are awesome");
exit();
```
